# (Perchlorato-κ*O*)tris­(triphenyl­phosphine-κ*P*)silver(I)

**DOI:** 10.1107/S1600536810025171

**Published:** 2010-07-03

**Authors:** Li-Na Cui, Ke-Yi Hu, Qiong-Hua Jin, Cun-Lin Zhang

**Affiliations:** a Department of Chemistry, Capital Normal University, Beijing 100048, People’s Republic of China; b Beijing Key Laboratory for Terahertz Spectroscopy and Imaging, Key Laboratory of Terahertz Optoelectronics, Ministry of Education, Capital Normal University, Beijing 100048, People’s Republic of China

## Abstract

In the title complex, [Ag(C_18_H_15_P)_3_(ClO_4_)], the silver coord­ination environment is dominated by the distorted P_3_AgO tetra­hedron in which Ag—O = 2.608 (12) Å and the Ag—P bond lengths are 2.5663 (17), 2.5076(16) and 2.5450 (17) Å. The perchlorate O-atoms are disordered over two positions in a 0.584 (14):0.416 (14) ratio.

## Related literature

For similar compounds, see: Awaleh *et al.* (2005 ▶); Effendy *et al.* (2007*a* ▶,*b* ▶,*c* ▶); Jin *et al.* (2010 ▶); Di Nicola *et al.* (2007 ▶); Pettinari *et al.* (2007 ▶). 
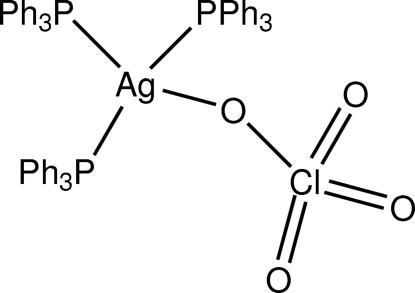


## Experimental

### 

#### Crystal data

[Ag(C_18_H_15_P)_3_(ClO_4_)]
*M* *_r_* = 994.13
Monoclinic, 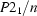

*a* = 18.066 (2) Å
*b* = 13.7779 (16) Å
*c* = 19.064 (2) Å
β = 94.132 (2)°
*V* = 4733.0 (9) Å^3^
*Z* = 4
Mo *K*α radiation
μ = 0.63 mm^−1^
*T* = 298 K
0.19 × 0.18 × 0.17 mm

#### Data collection

Bruker SMART CCD area-detector diffractometer
Absorption correction: multi-scan (*SADABS*; Sheldrick, 2008 ▶) *T*_min_ = 0.890, *T*_max_ = 0.901
23792 measured reflections
8339 independent reflections
4010 reflections with *I* > 2σ(*I*)
*R*_int_ = 0.069

#### Refinement

*R*[*F* ^2^ > 2σ(*F* ^2^)] = 0.057
*wR*(*F* ^2^) = 0.128
*S* = 1.03
8339 reflections
605 parameters
H-atom parameters constrained
Δρ_max_ = 0.85 e Å^−3^
Δρ_min_ = −1.07 e Å^−3^

### 

Data collection: *SMART* (Bruker, 2007 ▶); cell refinement: *SAINT* (Bruker, 2007 ▶); data reduction: *SAINT*; program(s) used to solve structure: *SHELXS97* (Sheldrick, 2008 ▶); program(s) used to refine structure: *SHELXL97* (Sheldrick, 2008 ▶); molecular graphics: *SHELXTL* (Sheldrick, 2008 ▶); software used to prepare material for publication: *SHELXTL*.

## Supplementary Material

Crystal structure: contains datablocks global, I. DOI: 10.1107/S1600536810025171/su2189sup1.cif

Structure factors: contains datablocks I. DOI: 10.1107/S1600536810025171/su2189Isup2.hkl

Additional supplementary materials:  crystallographic information; 3D view; checkCIF report

## Figures and Tables

**Table 1:** Selected bond angles (°)

P2—Ag1—P3	118.38 (6)
P2—Ag1—P1	114.66 (5)
P3—Ag1—P1	119.17 (6)
P2—Ag1—O1	92.9 (4)
P3—Ag1—O1	87.0 (4)
P1—Ag1—O1	118.0 (4)

**Fig. 1.**
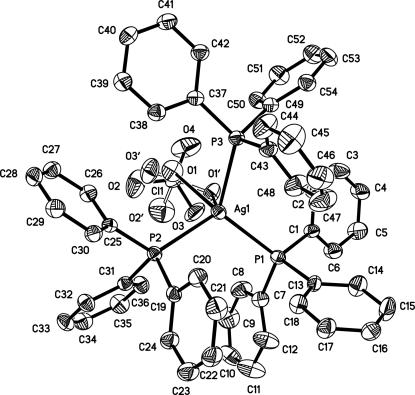
A view of a basic unit of the title complex. Displacement ellipsoids are drawn at the 50% probability level and H-atoms have been omitted for clarity.

## supplementary crystallographic information

### Comment

Reports concerning with the structural and kinetic features of silver(I)-phosphine complexes are growing in number as the participation of these compounds in biological processes are discovered. A recent report (Effendy *et al.*, 2007a) described a series of complexes of silver perchlorate and nitrate, tertiary phosphine ligand and oligodentate N-base adducts of the form AgX:dpex:*L*(2:1:2). In these complexes, the perchlorate acts either as a counteranion or as a ligand coordinating to silver(I) by one oxygen atom in a monodentate mode or by two oxygen atoms in a bidentate-chelating mode. We have studied the catalytic function of some nitrogen heterocyclic ligands (Jin *et al.*, 2010), and herein we report on the new title complex, which was synthesized under catalysis of 2-aminopyrimidine.

The molecular structure of the title complex is depicted in Fig. 1. The silver atom is four coordinated by three phosphorus atoms from PPh_3_ groups and one oxygen atom from the ClO_4_ anion. The Ag—P distances are 2.5641 (14), 2.5047 (13) Å and 2.5415 (13) Å. The Ag—O distance [2.668 (14) Å] is slightly longer than that in adducts of AgClO_4_:dppe:bpy [2.539 (2) Å] (Effendy *et al.*, 2007*a*). The angles P—Ag—P are in the range of 114.70 (4) - 119.17 (5)°, while angles P—Ag—O are in the range of 87.1 (4) - 118.1 (4) °, which comfirms the distorted tetrahedral environment around the silver atom.

The crystal structure data show little differences between the title complex and other Ag*X*:PPh_3_:*L*, where *X* is a simple inorganic or organic anion, for example, nitrate (Di Nicola *et al.*, 2007), nitrite (Pettinari *et al.*, 2007), acetate (Effendy *et al.*, 2007*b*), perchlorate (Effendy *et al.*, 2007*c*) or trifluoroacetate (Awaleh *et al.*, 2005).

### Experimental

A mixture of AgClO_4_, PPh_3_ and 2-aminopyrimidine, in the molar ratio 1:1:2, in CH_2_Cl_2_ and MeOH (10 ml, V/V=1/1) was stirred for 2 h at ambient temperature, then filtered. Subsequent slow evaporation of the filtrate resulted in the formation of colorless crystals of the title complex. Crystals suitable for single-crystal X-ray diffraction were selected directly from the sample as prepared. Analysis found: C 65.40%, H 5.07%; calculated: C 65.19%, H 4.35%.

### Refinement

The perchlorate O-atoms are disordered over two positions: refined occupancies O/O' = 0.584 (14)/0.416 (14). The H-atoms were included in calculated positions and treated as riding atoms: C-H = 0.93 Å with U_iso_(H) = 1.2U_eq_(parent C-atom).

### Crystal data

**Table:**

[Ag(C_18_H_15_P)_3_(ClO_4_)]	*F*(000) = 2040
*M**_r_* = 994.13	*D*_x_ = 1.395 Mg m^−^^3^
Monoclinic, *P*2_1_/*n*	Mo *K*α radiation, λ = 0.71073 Å
Hall symbol: -P 2yn	Cell parameters from 2750 reflections
*a* = 18.066 (2) Å	θ = 2.7–19.5°
*b* = 13.7779 (16) Å	µ = 0.63 mm^−^^1^
*c* = 19.064 (2) Å	*T* = 298 K
β = 94.132 (2)°	Block, colourless
*V* = 4733.0 (9) Å^3^	0.19 × 0.18 × 0.17 mm
*Z* = 4	

### Data collection

**Table:**

Bruker SMART CCD area-detector diffractometer	8339 independent reflections
Radiation source: fine-focus sealed tube	4010 reflections with *I* > 2σ(*I*)
graphite	*R*_int_ = 0.069
phi and ω scans	θ_max_ = 25.0°, θ_min_ = 1.5°
Absorption correction: multi-scan (*SADABS*; Sheldrick, 2008)	*h* = −21→19
*T*_min_ = 0.890, *T*_max_ = 0.901	*k* = −13→16
23792 measured reflections	*l* = −22→22

### Refinement

**Table:**

Refinement on *F*^2^	Primary atom site location: structure-invariant direct methods
Least-squares matrix: full	Secondary atom site location: difference Fourier map
*R*[*F*^2^ > 2σ(*F*^2^)] = 0.057	Hydrogen site location: inferred from neighbouring sites
*wR*(*F*^2^) = 0.128	H-atom parameters constrained
*S* = 1.03	*w* = 1/[σ^2^(*F*_o_^2^) + (0.0375*P*)^2^] where *P* = (*F*_o_^2^ + 2*F*_c_^2^)/3
8339 reflections	(Δ/σ)_max_ = 0.001
605 parameters	Δρ_max_ = 0.85 e Å^−^^3^
0 restraints	Δρ_min_ = −1.07 e Å^−^^3^

### Special details

**Table:**

Geometry. All e.s.d.'s (except the e.s.d. in the dihedral angle between two l.s. planes) are estimated using the full covariance matrix. The cell e.s.d.'s are taken into account individually in the estimation of e.s.d.'s in distances, angles and torsion angles; correlations between e.s.d.'s in cell parameters are only used when they are defined by crystal symmetry. An approximate (isotropic) treatment of cell e.s.d.'s is used for estimating e.s.d.'s involving l.s. planes.
Refinement. Refinement of *F*^2^ against ALL reflections. The weighted *R*-factor *wR* and goodness of fit *S* are based on *F*^2^, conventional *R*-factors *R* are based on *F*, with *F* set to zero for negative *F*^2^. The threshold expression of *F*^2^ > σ(*F*^2^) is used only for calculating *R*-factors(gt) *etc*. and is not relevant to the choice of reflections for refinement. *R*-factors based on *F*^2^ are statistically about twice as large as those based on *F*, and *R*- factors based on ALL data will be even larger.

### Fractional atomic coordinates and isotropic or equivalent isotropic displacement parameters (Å^2^)

**Table:**

	*x*	*y*	*z*	*U*_iso_*/*U*_eq_	Occ. (<1)
Ag1	0.24335 (3)	0.81327 (3)	0.01311 (2)	0.05196 (17)	
P1	0.34225 (9)	0.68176 (12)	0.00117 (8)	0.0493 (4)	
P2	0.17377 (9)	0.80044 (11)	0.12190 (8)	0.0477 (4)	
P3	0.26351 (10)	0.98099 (11)	−0.03809 (8)	0.0519 (5)	
Cl1	0.09855 (15)	0.72401 (17)	−0.11116 (12)	0.0802 (6)	
O1	0.1224 (7)	0.8041 (13)	−0.0700 (9)	0.089 (5)	0.584 (14)
O2	0.0255 (6)	0.7017 (6)	−0.0998 (4)	0.080 (4)	0.584 (14)
O3	0.1416 (7)	0.6407 (8)	−0.0977 (5)	0.080 (4)	0.584 (14)
O4	0.1075 (10)	0.7556 (15)	−0.1821 (10)	0.105 (6)	0.584 (14)
O1'	0.1765 (8)	0.7147 (13)	−0.0977 (7)	0.097 (6)	0.416 (14)
O2'	0.0779 (9)	0.6317 (10)	−0.0900 (7)	0.080 (6)	0.416 (14)
O3'	0.0800 (10)	0.8015 (17)	−0.0690 (12)	0.080 (6)	0.416 (14)
O4'	0.0664 (14)	0.728 (2)	−0.1794 (14)	0.105 (9)	0.416 (14)
C1	0.3718 (4)	0.6630 (5)	−0.0865 (3)	0.0579 (18)	
C2	0.3609 (4)	0.7343 (5)	−0.1358 (4)	0.084 (2)	
H2	0.3376	0.7916	−0.1241	0.101*	
C3	0.3840 (5)	0.7227 (6)	−0.2031 (4)	0.102 (3)	
H3	0.3756	0.7721	−0.2360	0.123*	
C4	0.4186 (5)	0.6403 (6)	−0.2217 (4)	0.088 (2)	
H4	0.4355	0.6342	−0.2664	0.106*	
C5	0.4285 (4)	0.5664 (6)	−0.1743 (4)	0.095 (3)	
H5	0.4508	0.5088	−0.1870	0.114*	
C6	0.4050 (4)	0.5775 (5)	−0.1071 (4)	0.082 (2)	
H6	0.4116	0.5267	−0.0751	0.099*	
C7	0.3143 (4)	0.5609 (5)	0.0251 (4)	0.0632 (19)	
C8	0.2590 (5)	0.5177 (6)	−0.0159 (4)	0.096 (3)	
H8	0.2371	0.5520	−0.0540	0.115*	
C9	0.2338 (5)	0.4246 (7)	−0.0034 (5)	0.117 (3)	
H9	0.1987	0.3953	−0.0346	0.141*	
C10	0.2612 (6)	0.3772 (7)	0.0549 (5)	0.111 (3)	
H10	0.2400	0.3187	0.0675	0.133*	
C11	0.3199 (7)	0.4149 (7)	0.0955 (5)	0.127 (4)	
H11	0.3425	0.3790	0.1324	0.153*	
C12	0.3457 (5)	0.5080 (6)	0.0809 (4)	0.100 (3)	
H12	0.3846	0.5345	0.1092	0.120*	
C13	0.4285 (4)	0.6985 (5)	0.0558 (3)	0.0600 (18)	
C14	0.4977 (4)	0.6821 (5)	0.0321 (4)	0.083 (2)	
H14	0.5015	0.6629	−0.0142	0.100*	
C15	0.5622 (4)	0.6935 (6)	0.0762 (5)	0.098 (3)	
H15	0.6085	0.6830	0.0594	0.117*	
C16	0.5561 (5)	0.7207 (5)	0.1453 (4)	0.079 (2)	
H16	0.5985	0.7264	0.1757	0.095*	
C17	0.4898 (5)	0.7387 (5)	0.1686 (4)	0.081 (2)	
H17	0.4861	0.7583	0.2149	0.097*	
C18	0.4253 (4)	0.7284 (5)	0.1235 (4)	0.072 (2)	
H18	0.3794	0.7422	0.1403	0.086*	
C19	0.2416 (3)	0.7986 (5)	0.1960 (3)	0.0549 (17)	
C20	0.2915 (4)	0.8770 (5)	0.2022 (3)	0.069 (2)	
H20	0.2876	0.9272	0.1695	0.083*	
C21	0.3461 (4)	0.8798 (6)	0.2567 (4)	0.087 (2)	
H21	0.3778	0.9329	0.2618	0.105*	
C22	0.3534 (4)	0.8046 (7)	0.3030 (4)	0.086 (2)	
H22	0.3909	0.8065	0.3390	0.104*	
C23	0.3075 (5)	0.7275 (6)	0.2978 (4)	0.087 (2)	
H23	0.3137	0.6770	0.3301	0.105*	
C24	0.2503 (4)	0.7230 (5)	0.2438 (4)	0.071 (2)	
H24	0.2186	0.6698	0.2402	0.085*	
C25	0.1121 (3)	0.8980 (4)	0.1440 (3)	0.0477 (16)	
C26	0.0521 (4)	0.9169 (4)	0.0966 (3)	0.0610 (18)	
H26	0.0459	0.8811	0.0552	0.073*	
C27	0.0013 (4)	0.9883 (5)	0.1101 (4)	0.070 (2)	
H27	−0.0393	0.9993	0.0783	0.084*	
C28	0.0105 (4)	1.0430 (5)	0.1701 (4)	0.069 (2)	
H28	−0.0237	1.0910	0.1791	0.083*	
C29	0.0696 (4)	1.0266 (5)	0.2160 (4)	0.069 (2)	
H29	0.0767	1.0650	0.2561	0.082*	
C30	0.1202 (4)	0.9529 (5)	0.2045 (3)	0.0609 (18)	
H30	0.1594	0.9408	0.2376	0.073*	
C31	0.1149 (3)	0.6960 (4)	0.1318 (3)	0.0524 (16)	
C32	0.0666 (4)	0.6891 (5)	0.1848 (4)	0.0699 (19)	
H32	0.0660	0.7379	0.2185	0.084*	
C33	0.0191 (4)	0.6104 (6)	0.1885 (4)	0.082 (2)	
H33	−0.0127	0.6062	0.2246	0.098*	
C34	0.0192 (4)	0.5392 (6)	0.1388 (5)	0.082 (2)	
H34	−0.0135	0.4872	0.1405	0.099*	
C35	0.0671 (5)	0.5438 (5)	0.0865 (4)	0.088 (2)	
H35	0.0678	0.4944	0.0534	0.105*	
C36	0.1148 (4)	0.6225 (5)	0.0831 (4)	0.070 (2)	
H36	0.1471	0.6254	0.0474	0.083*	
C37	0.1963 (4)	1.0789 (4)	−0.0370 (3)	0.0534 (17)	
C38	0.1552 (4)	1.0904 (5)	0.0203 (4)	0.0644 (19)	
H38	0.1614	1.0470	0.0576	0.077*	
C39	0.1041 (4)	1.1663 (5)	0.0236 (4)	0.070 (2)	
H39	0.0768	1.1730	0.0628	0.084*	
C40	0.0943 (4)	1.2308 (5)	−0.0310 (4)	0.067 (2)	
H40	0.0610	1.2820	−0.0286	0.080*	
C41	0.1332 (4)	1.2192 (5)	−0.0882 (4)	0.074 (2)	
H41	0.1254	1.2619	−0.1257	0.089*	
C42	0.1845 (4)	1.1457 (5)	−0.0922 (3)	0.0660 (19)	
H42	0.2114	1.1403	−0.1318	0.079*	
C43	0.3407 (4)	1.0289 (5)	0.0146 (3)	0.0617 (19)	
C44	0.3482 (5)	1.1219 (6)	0.0385 (4)	0.103 (3)	
H44	0.3118	1.1669	0.0242	0.123*	
C45	0.4078 (6)	1.1526 (7)	0.0834 (5)	0.121 (3)	
H45	0.4110	1.2169	0.0982	0.145*	
C46	0.4611 (5)	1.0881 (8)	0.1054 (4)	0.109 (3)	
H46	0.5006	1.1077	0.1361	0.131*	
C47	0.4567 (5)	0.9952 (8)	0.0826 (5)	0.127 (4)	
H47	0.4937	0.9508	0.0964	0.152*	
C48	0.3963 (5)	0.9671 (6)	0.0385 (4)	0.101 (3)	
H48	0.3933	0.9026	0.0242	0.121*	
C49	0.2882 (4)	0.9838 (4)	−0.1286 (3)	0.0586 (18)	
C50	0.2438 (4)	0.9347 (5)	−0.1780 (4)	0.073 (2)	
H50	0.2044	0.8975	−0.1641	0.088*	
C51	0.2574 (5)	0.9402 (6)	−0.2484 (4)	0.089 (2)	
H51	0.2264	0.9072	−0.2814	0.106*	
C52	0.3145 (5)	0.9924 (6)	−0.2702 (4)	0.091 (3)	
H52	0.3232	0.9946	−0.3177	0.109*	
C53	0.3594 (4)	1.0416 (5)	−0.2222 (4)	0.083 (2)	
H53	0.3988	1.0780	−0.2368	0.099*	
C54	0.3465 (4)	1.0378 (5)	−0.1515 (4)	0.071 (2)	
H54	0.3774	1.0718	−0.1190	0.086*	

### Atomic displacement parameters (Å^2^)

**Table:**

	*U*^11^	*U*^22^	*U*^33^	*U*^12^	*U*^13^	*U*^23^
Ag1	0.0555 (3)	0.0565 (3)	0.0441 (3)	−0.0045 (3)	0.0050 (2)	−0.0010 (3)
P1	0.0562 (11)	0.0492 (10)	0.0425 (9)	−0.0009 (9)	0.0046 (8)	−0.0011 (8)
P2	0.0503 (11)	0.0525 (11)	0.0405 (9)	−0.0021 (8)	0.0053 (8)	−0.0001 (8)
P3	0.0540 (12)	0.0500 (11)	0.0511 (10)	−0.0032 (8)	−0.0011 (9)	0.0055 (8)
Cl1	0.0718 (19)	0.0748 (17)	0.0907 (17)	0.0112 (14)	−0.0162 (14)	−0.0081 (13)
O1	0.066 (10)	0.110 (9)	0.087 (7)	−0.031 (11)	−0.029 (9)	−0.029 (6)
O2	0.072 (8)	0.075 (7)	0.091 (7)	0.011 (5)	−0.016 (5)	−0.008 (5)
O3	0.072 (9)	0.075 (8)	0.091 (7)	0.011 (7)	−0.016 (6)	−0.008 (5)
O4	0.126 (16)	0.123 (13)	0.058 (8)	0.007 (10)	−0.042 (11)	−0.014 (7)
O1'	0.072 (11)	0.097 (14)	0.116 (11)	0.001 (9)	−0.028 (8)	−0.033 (9)
O2'	0.072 (13)	0.075 (11)	0.091 (10)	0.011 (9)	−0.016 (9)	−0.008 (7)
O3'	0.072 (13)	0.075 (12)	0.091 (10)	0.011 (12)	−0.016 (12)	−0.008 (8)
O4'	0.13 (2)	0.123 (18)	0.058 (11)	0.007 (14)	−0.042 (15)	−0.014 (10)
C1	0.072 (5)	0.058 (5)	0.045 (4)	0.008 (4)	0.011 (3)	−0.002 (3)
C2	0.114 (7)	0.081 (6)	0.060 (5)	0.024 (5)	0.021 (5)	−0.005 (4)
C3	0.151 (9)	0.099 (7)	0.060 (5)	0.025 (6)	0.026 (5)	0.016 (5)
C4	0.120 (7)	0.093 (6)	0.056 (5)	0.020 (5)	0.036 (5)	0.001 (5)
C5	0.122 (8)	0.100 (7)	0.066 (5)	0.036 (5)	0.033 (5)	−0.007 (5)
C6	0.105 (7)	0.082 (6)	0.062 (5)	0.025 (5)	0.020 (5)	0.003 (4)
C7	0.084 (6)	0.053 (5)	0.053 (4)	0.006 (4)	0.010 (4)	−0.004 (4)
C8	0.099 (7)	0.072 (6)	0.113 (7)	−0.011 (5)	−0.020 (6)	0.009 (5)
C9	0.130 (9)	0.082 (8)	0.139 (9)	−0.028 (6)	0.002 (7)	−0.006 (6)
C10	0.148 (10)	0.078 (7)	0.111 (8)	−0.039 (7)	0.037 (7)	0.001 (6)
C11	0.195 (12)	0.096 (8)	0.090 (7)	−0.023 (7)	0.006 (7)	0.009 (6)
C12	0.150 (9)	0.072 (6)	0.075 (6)	−0.030 (6)	−0.009 (6)	0.003 (5)
C13	0.057 (5)	0.067 (5)	0.055 (4)	0.010 (4)	0.002 (4)	0.001 (4)
C14	0.075 (6)	0.111 (6)	0.062 (5)	0.012 (5)	−0.004 (4)	−0.016 (5)
C15	0.059 (6)	0.133 (7)	0.100 (7)	0.012 (5)	0.000 (5)	−0.006 (6)
C16	0.066 (6)	0.095 (6)	0.074 (6)	0.002 (4)	−0.019 (5)	0.003 (4)
C17	0.072 (6)	0.101 (6)	0.068 (5)	0.002 (5)	−0.009 (5)	−0.006 (4)
C18	0.068 (6)	0.083 (5)	0.064 (5)	0.005 (4)	0.000 (4)	−0.014 (4)
C19	0.055 (5)	0.066 (5)	0.044 (4)	0.003 (4)	0.008 (3)	−0.001 (3)
C20	0.065 (5)	0.082 (5)	0.058 (5)	−0.006 (4)	−0.004 (4)	−0.001 (4)
C21	0.077 (6)	0.109 (7)	0.074 (6)	−0.010 (5)	−0.011 (5)	−0.016 (5)
C22	0.074 (6)	0.120 (8)	0.062 (5)	0.014 (6)	−0.013 (4)	−0.005 (5)
C23	0.093 (7)	0.102 (7)	0.066 (5)	0.013 (5)	−0.004 (5)	0.005 (5)
C24	0.068 (5)	0.083 (6)	0.062 (5)	0.010 (4)	0.000 (4)	0.001 (4)
C25	0.052 (5)	0.045 (4)	0.046 (4)	−0.004 (3)	0.007 (3)	0.002 (3)
C26	0.065 (5)	0.058 (5)	0.059 (4)	0.001 (4)	0.000 (4)	−0.006 (3)
C27	0.064 (5)	0.069 (5)	0.077 (5)	0.001 (4)	0.002 (4)	0.003 (4)
C28	0.073 (6)	0.059 (5)	0.078 (5)	0.007 (4)	0.022 (4)	0.000 (4)
C29	0.084 (6)	0.063 (5)	0.060 (5)	0.004 (4)	0.013 (4)	−0.008 (4)
C30	0.068 (5)	0.062 (5)	0.053 (4)	0.005 (4)	0.007 (4)	0.002 (4)
C31	0.054 (4)	0.052 (4)	0.051 (4)	0.003 (3)	0.003 (3)	0.004 (3)
C32	0.078 (5)	0.062 (5)	0.071 (5)	−0.008 (4)	0.015 (4)	−0.002 (4)
C33	0.076 (6)	0.075 (6)	0.097 (6)	−0.013 (5)	0.025 (5)	0.018 (5)
C34	0.079 (7)	0.065 (6)	0.101 (7)	−0.017 (4)	−0.004 (5)	0.012 (5)
C35	0.096 (7)	0.066 (6)	0.100 (7)	−0.011 (5)	0.001 (6)	−0.010 (5)
C36	0.073 (6)	0.063 (5)	0.074 (5)	−0.007 (4)	0.013 (4)	−0.002 (4)
C37	0.059 (5)	0.048 (4)	0.052 (4)	−0.007 (3)	−0.003 (4)	0.001 (3)
C38	0.067 (5)	0.060 (5)	0.066 (5)	−0.002 (4)	0.001 (4)	0.002 (4)
C39	0.072 (6)	0.065 (5)	0.071 (5)	−0.001 (4)	0.005 (4)	−0.006 (4)
C40	0.067 (5)	0.059 (5)	0.072 (5)	0.009 (4)	−0.003 (4)	−0.007 (4)
C41	0.087 (6)	0.064 (5)	0.071 (5)	0.012 (4)	−0.004 (5)	0.010 (4)
C42	0.073 (5)	0.065 (5)	0.060 (5)	0.007 (4)	0.005 (4)	0.004 (4)
C43	0.062 (5)	0.061 (5)	0.061 (4)	−0.004 (4)	−0.007 (4)	0.014 (4)
C44	0.085 (7)	0.095 (7)	0.123 (8)	−0.004 (5)	−0.024 (6)	−0.014 (6)
C45	0.108 (9)	0.109 (8)	0.140 (9)	−0.016 (6)	−0.028 (7)	−0.040 (7)
C46	0.096 (8)	0.128 (9)	0.098 (7)	−0.027 (7)	−0.037 (6)	0.006 (6)
C47	0.098 (8)	0.125 (9)	0.149 (9)	−0.010 (7)	−0.050 (7)	0.025 (7)
C48	0.086 (7)	0.088 (7)	0.122 (7)	−0.012 (5)	−0.031 (6)	0.002 (5)
C49	0.066 (5)	0.054 (4)	0.055 (4)	0.001 (4)	−0.002 (4)	0.006 (3)
C50	0.080 (6)	0.079 (5)	0.062 (5)	−0.014 (4)	0.007 (4)	0.001 (4)
C51	0.097 (7)	0.104 (6)	0.063 (5)	−0.010 (5)	−0.011 (5)	−0.003 (5)
C52	0.104 (8)	0.103 (7)	0.067 (6)	−0.001 (5)	0.011 (6)	0.006 (5)
C53	0.083 (6)	0.092 (6)	0.076 (6)	−0.009 (5)	0.025 (5)	0.017 (5)
C54	0.073 (6)	0.076 (5)	0.066 (5)	−0.007 (4)	0.005 (4)	0.005 (4)

### Geometric parameters (Å, °)

**Table:**

Ag1—P2	2.5076 (16)	C22—C23	1.347 (9)
Ag1—P3	2.5450 (17)	C22—H22	0.9300
Ag1—P1	2.5663 (17)	C23—C24	1.407 (9)
Ag1—O1	2.608 (12)	C23—H23	0.9300
P1—C7	1.809 (7)	C24—H24	0.9300
P1—C1	1.809 (6)	C25—C30	1.378 (7)
P1—C13	1.826 (6)	C25—C26	1.386 (7)
P2—C19	1.803 (6)	C26—C27	1.382 (8)
P2—C31	1.808 (6)	C26—H26	0.9300
P2—C25	1.815 (6)	C27—C28	1.369 (8)
P3—C43	1.785 (7)	C27—H27	0.9300
P3—C49	1.814 (7)	C28—C29	1.351 (8)
P3—C37	1.815 (6)	C28—H28	0.9300
Cl1—O2	1.386 (10)	C29—C30	1.393 (8)
Cl1—O4'	1.39 (2)	C29—H29	0.9300
Cl1—O3'	1.39 (2)	C30—H30	0.9300
Cl1—O2'	1.393 (14)	C31—C36	1.373 (8)
Cl1—O3	1.400 (10)	C31—C32	1.386 (8)
Cl1—O1	1.403 (16)	C32—C33	1.388 (9)
Cl1—O1'	1.419 (14)	C32—H32	0.9300
Cl1—O4	1.44 (2)	C33—C34	1.363 (9)
C1—C2	1.364 (8)	C33—H33	0.9300
C1—C6	1.391 (8)	C34—C35	1.368 (10)
C2—C3	1.388 (9)	C34—H34	0.9300
C2—H2	0.9300	C35—C36	1.389 (9)
C3—C4	1.354 (9)	C35—H35	0.9300
C3—H3	0.9300	C36—H36	0.9300
C4—C5	1.365 (9)	C37—C38	1.374 (8)
C4—H4	0.9300	C37—C42	1.403 (8)
C5—C6	1.386 (9)	C38—C39	1.399 (8)
C5—H5	0.9300	C38—H38	0.9300
C6—H6	0.9300	C39—C40	1.369 (8)
C7—C8	1.361 (9)	C39—H39	0.9300
C7—C12	1.377 (9)	C40—C41	1.349 (9)
C8—C9	1.386 (10)	C40—H40	0.9300
C8—H8	0.9300	C41—C42	1.378 (8)
C9—C10	1.351 (11)	C41—H41	0.9300
C9—H9	0.9300	C42—H42	0.9300
C10—C11	1.370 (11)	C43—C44	1.363 (9)
C10—H10	0.9300	C43—C48	1.370 (9)
C11—C12	1.399 (10)	C44—C45	1.392 (10)
C11—H11	0.9300	C44—H44	0.9300
C12—H12	0.9300	C45—C46	1.354 (11)
C13—C18	1.359 (8)	C45—H45	0.9300
C13—C14	1.378 (9)	C46—C47	1.353 (11)
C14—C15	1.395 (9)	C46—H46	0.9300
C14—H14	0.9300	C47—C48	1.384 (10)
C15—C16	1.382 (9)	C47—H47	0.9300
C15—H15	0.9300	C48—H48	0.9300
C16—C17	1.330 (9)	C49—C50	1.370 (8)
C16—H16	0.9300	C49—C54	1.386 (8)
C17—C18	1.405 (8)	C50—C51	1.385 (9)
C17—H17	0.9300	C50—H50	0.9300
C18—H18	0.9300	C51—C52	1.348 (10)
C19—C24	1.385 (8)	C51—H51	0.9300
C19—C20	1.406 (8)	C52—C53	1.360 (9)
C20—C21	1.382 (8)	C52—H52	0.9300
C20—H20	0.9300	C53—C54	1.384 (8)
C21—C22	1.360 (9)	C53—H53	0.9300
C21—H21	0.9300	C54—H54	0.9300
			
P2—Ag1—P3	118.38 (6)	C24—C19—P2	124.3 (5)
P2—Ag1—P1	114.66 (5)	C20—C19—P2	116.7 (5)
P3—Ag1—P1	119.17 (6)	C21—C20—C19	120.2 (7)
P2—Ag1—O1	92.9 (4)	C21—C20—H20	119.9
P3—Ag1—O1	87.0 (4)	C19—C20—H20	119.9
P1—Ag1—O1	118.0 (4)	C22—C21—C20	119.8 (7)
C7—P1—C1	102.0 (3)	C22—C21—H21	120.1
C7—P1—C13	102.4 (3)	C20—C21—H21	120.1
C1—P1—C13	104.4 (3)	C23—C22—C21	121.5 (8)
C7—P1—Ag1	114.7 (2)	C23—C22—H22	119.2
C1—P1—Ag1	115.9 (2)	C21—C22—H22	119.2
C13—P1—Ag1	115.6 (2)	C22—C23—C24	120.2 (8)
C19—P2—C31	106.2 (3)	C22—C23—H23	119.9
C19—P2—C25	102.8 (3)	C24—C23—H23	119.9
C31—P2—C25	101.0 (3)	C19—C24—C23	119.4 (7)
C19—P2—Ag1	107.2 (2)	C19—C24—H24	120.3
C31—P2—Ag1	118.4 (2)	C23—C24—H24	120.3
C25—P2—Ag1	119.49 (19)	C30—C25—C26	118.2 (6)
C43—P3—C49	107.0 (3)	C30—C25—P2	124.9 (5)
C43—P3—C37	102.4 (3)	C26—C25—P2	116.9 (5)
C49—P3—C37	101.8 (3)	C27—C26—C25	120.7 (6)
C43—P3—Ag1	104.3 (2)	C27—C26—H26	119.6
C49—P3—Ag1	115.9 (2)	C25—C26—H26	119.6
C37—P3—Ag1	123.8 (2)	C28—C27—C26	120.4 (7)
O2—Cl1—O4'	79.4 (12)	C28—C27—H27	119.8
O2—Cl1—O3'	79.0 (9)	C26—C27—H27	119.8
O4'—Cl1—O3'	113.9 (16)	C29—C28—C27	119.5 (7)
O2—Cl1—O2'	58.4 (7)	C29—C28—H28	120.3
O4'—Cl1—O2'	102.1 (12)	C27—C28—H28	120.3
O3'—Cl1—O2'	116.9 (11)	C28—C29—C30	121.0 (6)
O2—Cl1—O3	108.2 (7)	C28—C29—H29	119.5
O4'—Cl1—O3	113.4 (14)	C30—C29—H29	119.5
O3'—Cl1—O3	132.6 (9)	C25—C30—C29	120.1 (6)
O2'—Cl1—O3	49.8 (6)	C25—C30—H30	119.9
O2—Cl1—O1	110.2 (7)	C29—C30—H30	119.9
O4'—Cl1—O1	125.5 (14)	C36—C31—C32	118.1 (6)
O3'—Cl1—O1	31.9 (7)	C36—C31—P2	119.3 (5)
O2'—Cl1—O1	129.3 (9)	C32—C31—P2	122.5 (5)
O3—Cl1—O1	113.5 (8)	C31—C32—C33	121.0 (7)
O2—Cl1—O1'	153.5 (8)	C31—C32—H32	119.5
O4'—Cl1—O1'	121.1 (14)	C33—C32—H32	119.5
O3'—Cl1—O1'	104.0 (8)	C34—C33—C32	119.7 (8)
O2'—Cl1—O1'	98.5 (9)	C34—C33—H33	120.1
O3—Cl1—O1'	50.4 (7)	C32—C33—H33	120.1
O1—Cl1—O1'	73.1 (7)	C33—C34—C35	120.4 (7)
O2—Cl1—O4	112.7 (7)	C33—C34—H34	119.8
O4'—Cl1—O4	34.4 (10)	C35—C34—H34	119.8
O3'—Cl1—O4	111.0 (13)	C34—C35—C36	119.8 (7)
O2'—Cl1—O4	126.7 (9)	C34—C35—H35	120.1
O3—Cl1—O4	108.7 (11)	C36—C35—H35	120.1
O1—Cl1—O4	103.7 (11)	C31—C36—C35	121.0 (7)
O1'—Cl1—O4	91.1 (10)	C31—C36—H36	119.5
Cl1—O1—Ag1	126.0 (10)	C35—C36—H36	119.5
C2—C1—C6	117.3 (6)	C38—C37—C42	117.2 (6)
C2—C1—P1	119.7 (5)	C38—C37—P3	119.7 (5)
C6—C1—P1	123.0 (5)	C42—C37—P3	123.0 (5)
C1—C2—C3	121.1 (7)	C37—C38—C39	121.2 (6)
C1—C2—H2	119.5	C37—C38—H38	119.4
C3—C2—H2	119.5	C39—C38—H38	119.4
C4—C3—C2	120.9 (7)	C40—C39—C38	120.1 (7)
C4—C3—H3	119.6	C40—C39—H39	120.0
C2—C3—H3	119.6	C38—C39—H39	120.0
C3—C4—C5	119.5 (7)	C41—C40—C39	119.4 (7)
C3—C4—H4	120.2	C41—C40—H40	120.3
C5—C4—H4	120.2	C39—C40—H40	120.3
C4—C5—C6	119.7 (7)	C40—C41—C42	121.6 (7)
C4—C5—H5	120.2	C40—C41—H41	119.2
C6—C5—H5	120.2	C42—C41—H41	119.2
C5—C6—C1	121.4 (7)	C41—C42—C37	120.5 (7)
C1—C6—H6	119.3	C41—C42—H42	119.8
C8—C7—C12	117.1 (7)	C37—C42—H42	119.8
C8—C7—P1	117.7 (6)	C44—C43—C48	114.9 (7)
C12—C7—P1	125.1 (6)	C44—C43—P3	126.2 (6)
C7—C8—C9	122.8 (8)	C48—C43—P3	118.8 (6)
C7—C8—H8	118.6	C43—C44—C45	123.0 (8)
C9—C8—H8	118.6	C43—C44—H44	118.5
C10—C9—C8	119.0 (9)	C45—C44—H44	118.5
C10—C9—H9	120.5	C46—C45—C44	119.5 (9)
C8—C9—H9	120.5	C46—C45—H45	120.2
C9—C10—C11	120.2 (9)	C44—C45—H45	120.2
C9—C10—H10	119.9	C47—C46—C45	119.8 (9)
C11—C10—H10	119.9	C47—C46—H46	120.1
C10—C11—C12	119.3 (9)	C45—C46—H46	120.1
C10—C11—H11	120.3	C46—C47—C48	119.1 (9)
C12—C11—H11	120.3	C46—C47—H47	120.5
C7—C12—C11	121.0 (8)	C48—C47—H47	120.5
C7—C12—H12	119.5	C43—C48—C47	123.7 (8)
C11—C12—H12	119.5	C43—C48—H48	118.1
C18—C13—C14	117.6 (6)	C47—C48—H48	118.1
C18—C13—P1	119.1 (6)	C50—C49—C54	118.0 (6)
C14—C13—P1	123.4 (5)	C50—C49—P3	118.1 (6)
C13—C14—C15	121.4 (7)	C54—C49—P3	123.8 (5)
C13—C14—H14	119.3	C49—C50—C51	120.2 (7)
C15—C14—H14	119.3	C49—C50—H50	119.9
C16—C15—C14	119.0 (8)	C51—C50—H50	119.9
C16—C15—H15	120.5	C52—C51—C50	121.4 (8)
C14—C15—H15	120.5	C52—C51—H51	119.3
C17—C16—C15	120.3 (7)	C50—C51—H51	119.3
C17—C16—H16	119.9	C51—C52—C53	119.4 (8)
C15—C16—H16	119.9	C51—C52—H52	120.3
C16—C17—C18	120.3 (7)	C53—C52—H52	120.3
C16—C17—H17	119.9	C52—C53—C54	120.2 (7)
C18—C17—H17	119.9	C52—C53—H53	119.9
C13—C18—C17	121.4 (7)	C54—C53—H53	119.9
C13—C18—H18	119.3	C53—C54—C49	120.8 (7)
C17—C18—H18	119.3	C53—C54—H54	119.6
C24—C19—C20	118.8 (6)	C49—C54—H54	119.6
			
P2—Ag1—P1—C7	41.9 (3)	C31—P2—C19—C20	−176.2 (5)
P3—Ag1—P1—C7	−169.3 (2)	C25—P2—C19—C20	−70.6 (5)
O1—Ag1—P1—C7	−66.1 (5)	Ag1—P2—C19—C20	56.2 (5)
P2—Ag1—P1—C1	160.5 (2)	C24—C19—C20—C21	−2.5 (10)
P3—Ag1—P1—C1	−50.7 (2)	P2—C19—C20—C21	−178.6 (5)
O1—Ag1—P1—C1	52.5 (5)	C19—C20—C21—C22	2.5 (11)
P2—Ag1—P1—C13	−77.0 (2)	C20—C21—C22—C23	−1.2 (13)
P3—Ag1—P1—C13	71.8 (2)	C21—C22—C23—C24	0.0 (13)
O1—Ag1—P1—C13	175.1 (5)	C20—C19—C24—C23	1.3 (10)
P3—Ag1—P2—C19	−94.4 (2)	P2—C19—C24—C23	177.0 (5)
P1—Ag1—P2—C19	54.6 (2)	C22—C23—C24—C19	0.0 (11)
O1—Ag1—P2—C19	177.4 (5)	C19—P2—C25—C30	−0.2 (6)
P3—Ag1—P2—C31	145.6 (2)	C31—P2—C25—C30	109.5 (6)
P1—Ag1—P2—C31	−65.4 (2)	Ag1—P2—C25—C30	−118.7 (5)
O1—Ag1—P2—C31	57.4 (4)	C19—P2—C25—C26	−179.1 (5)
P3—Ag1—P2—C25	21.9 (2)	C31—P2—C25—C26	−69.4 (5)
P1—Ag1—P2—C25	170.9 (2)	Ag1—P2—C25—C26	62.4 (5)
O1—Ag1—P2—C25	−66.3 (5)	C30—C25—C26—C27	−0.6 (10)
P2—Ag1—P3—C43	80.6 (3)	P2—C25—C26—C27	178.4 (5)
P1—Ag1—P3—C43	−67.0 (3)	C25—C26—C27—C28	1.3 (11)
O1—Ag1—P3—C43	172.3 (5)	C26—C27—C28—C29	0.0 (11)
P2—Ag1—P3—C49	−162.1 (2)	C27—C28—C29—C30	−2.0 (11)
P1—Ag1—P3—C49	50.3 (3)	C26—C25—C30—C29	−1.4 (9)
O1—Ag1—P3—C49	−70.4 (5)	P2—C25—C30—C29	179.8 (5)
P2—Ag1—P3—C37	−35.3 (2)	C28—C29—C30—C25	2.7 (11)
P1—Ag1—P3—C37	177.0 (2)	C19—P2—C31—C36	−114.7 (5)
O1—Ag1—P3—C37	56.4 (5)	C25—P2—C31—C36	138.3 (5)
O2—Cl1—O1—Ag1	129.0 (9)	Ag1—P2—C31—C36	5.8 (6)
O4'—Cl1—O1—Ag1	−139.7 (17)	C19—P2—C31—C32	68.7 (6)
O3'—Cl1—O1—Ag1	142 (3)	C25—P2—C31—C32	−38.3 (6)
O2'—Cl1—O1—Ag1	64.0 (16)	Ag1—P2—C31—C32	−170.8 (4)
O3—Cl1—O1—Ag1	7.6 (14)	C36—C31—C32—C33	−0.4 (10)
O1'—Cl1—O1—Ag1	−23.2 (10)	P2—C31—C32—C33	176.2 (5)
O4—Cl1—O1—Ag1	−110.2 (12)	C31—C32—C33—C34	−0.7 (11)
P2—Ag1—O1—Cl1	−106.1 (11)	C32—C33—C34—C35	1.6 (12)
P3—Ag1—O1—Cl1	135.6 (12)	C33—C34—C35—C36	−1.4 (12)
P1—Ag1—O1—Cl1	13.9 (13)	C32—C31—C36—C35	0.6 (10)
C7—P1—C1—C2	146.1 (6)	P2—C31—C36—C35	−176.2 (5)
C13—P1—C1—C2	−107.6 (6)	C34—C35—C36—C31	0.3 (11)
Ag1—P1—C1—C2	20.7 (7)	C43—P3—C37—C38	−79.7 (6)
C7—P1—C1—C6	−33.1 (7)	C49—P3—C37—C38	169.7 (5)
C13—P1—C1—C6	73.2 (7)	Ag1—P3—C37—C38	37.1 (6)
Ag1—P1—C1—C6	−158.5 (5)	C43—P3—C37—C42	99.3 (5)
C6—C1—C2—C3	−1.8 (11)	C49—P3—C37—C42	−11.3 (6)
P1—C1—C2—C3	179.0 (6)	Ag1—P3—C37—C42	−143.8 (4)
C1—C2—C3—C4	−0.5 (13)	C42—C37—C38—C39	−0.6 (9)
C2—C3—C4—C5	2.4 (14)	P3—C37—C38—C39	178.5 (5)
C3—C4—C5—C6	−1.9 (13)	C37—C38—C39—C40	0.1 (10)
C4—C5—C6—C1	−0.4 (12)	C38—C39—C40—C41	1.1 (10)
C2—C1—C6—C5	2.3 (11)	C39—C40—C41—C42	−1.9 (11)
P1—C1—C6—C5	−178.6 (6)	C40—C41—C42—C37	1.5 (11)
C1—P1—C7—C8	−59.7 (7)	C38—C37—C42—C41	−0.2 (9)
C13—P1—C7—C8	−167.6 (6)	P3—C37—C42—C41	−179.3 (5)
Ag1—P1—C7—C8	66.4 (6)	C49—P3—C43—C44	97.5 (7)
C1—P1—C7—C12	118.7 (7)	C37—P3—C43—C44	−9.2 (8)
C13—P1—C7—C12	10.9 (7)	Ag1—P3—C43—C44	−139.3 (6)
Ag1—P1—C7—C12	−115.1 (6)	C49—P3—C43—C48	−87.6 (6)
C12—C7—C8—C9	0.3 (13)	C37—P3—C43—C48	165.8 (6)
P1—C7—C8—C9	178.8 (7)	Ag1—P3—C43—C48	35.7 (7)
C7—C8—C9—C10	5.1 (15)	C48—C43—C44—C45	0.4 (12)
C8—C9—C10—C11	−8.9 (16)	P3—C43—C44—C45	175.5 (7)
C9—C10—C11—C12	7.4 (16)	C43—C44—C45—C46	−0.6 (15)
C8—C7—C12—C11	−1.8 (12)	C44—C45—C46—C47	1.3 (16)
P1—C7—C12—C11	179.7 (7)	C45—C46—C47—C48	−1.8 (16)
C10—C11—C12—C7	−1.9 (14)	C44—C43—C48—C47	−0.9 (13)
C7—P1—C13—C18	−83.5 (6)	P3—C43—C48—C47	−176.5 (7)
C1—P1—C13—C18	170.5 (5)	C46—C47—C48—C43	1.7 (15)
Ag1—P1—C13—C18	42.0 (6)	C43—P3—C49—C50	168.3 (5)
C7—P1—C13—C14	96.3 (6)	C37—P3—C49—C50	−84.6 (6)
C1—P1—C13—C14	−9.7 (7)	Ag1—P3—C49—C50	52.5 (6)
Ag1—P1—C13—C14	−138.2 (6)	C43—P3—C49—C54	−15.9 (7)
C18—C13—C14—C15	1.4 (11)	C37—P3—C49—C54	91.1 (6)
P1—C13—C14—C15	−178.4 (6)	Ag1—P3—C49—C54	−131.8 (5)
C13—C14—C15—C16	0.9 (12)	C54—C49—C50—C51	−0.5 (10)
C14—C15—C16—C17	−2.2 (12)	P3—C49—C50—C51	175.5 (6)
C15—C16—C17—C18	1.4 (12)	C49—C50—C51—C52	0.9 (12)
C14—C13—C18—C17	−2.3 (10)	C50—C51—C52—C53	−0.8 (13)
P1—C13—C18—C17	177.5 (5)	C51—C52—C53—C54	0.3 (13)
C16—C17—C18—C13	1.0 (11)	C52—C53—C54—C49	0.1 (11)
C31—P2—C19—C24	7.9 (6)	C50—C49—C54—C53	0.0 (10)
C25—P2—C19—C24	113.5 (6)	P3—C49—C54—C53	−175.8 (5)
Ag1—P2—C19—C24	−119.6 (5)		
